# *Lippia alba*—a potential bioresource for the management of *Spodoptera frugiperda* (Lepidoptera: Noctuidae)

**DOI:** 10.3389/fpls.2024.1422578

**Published:** 2024-08-08

**Authors:** Shreosi Biswas, Aditi Kundu, S. B. Suby, Abran Singh Kushwah, Neeraj Patanjali, Ajit Kumar Shasany, Rajesh Verma, Supradip Saha, Abhishek Mandal, Tirthankar Banerjee, Anil Kumar, Anupama Singh

**Affiliations:** ^1^ Division of Agricultural Chemicals, ICAR-Indian Agricultural Research Institute, New Delhi, India; ^2^ The Graduate School, ICAR-Indian Agricultural Research Institute, New Delhi, India; ^3^ ICAR-Indian Institute of Maize Research, Ludhiana, India; ^4^ CSIR-National Botanical Research Institute, Lucknow, Uttar Pradesh, India; ^5^ CSIR-Central Institute of Medicinal and Aromatic Plants, Lucknow, Uttar Pradesh, India; ^6^ ICAR-Indian Institute of Horticultural Research, Bengaluru, Karnataka, India; ^7^ Indian Council of Agricultural Research, New Delhi, India

**Keywords:** *Lippia*, *Spodoptera*, UPLC-QTOF-MS, *in silico*, botanicals

## Abstract

Fall armyworm (FAW), *Spodoptera frugiperda* (J.E. Smith), a threat to maize production systems, is a polyphagous pest of global significance. There is no registered bioinsecticide of botanical origin to provide green remedy against this pest of concern. The present study reports for the first time the potency of the polar and non-polar bioinsecticidal leads sourced from *Lippia alba* (Mill.) N.E. Br. leaves. Shade-dried leaves of *L*. *alba* were extracted and evaluated; based on preliminary bioassay, the ethyl acetate leaf extract of *L*. *alba* (*LEAE*) was found to be the most potent against FAW in the *in vitro* and *in vivo* studies. Ultraperformance liquid chromatography–quadrupole time-of-flight–mass spectrometric (UPLC-QToF-MS) analysis of *LEAE* revealed the rich chemical profile of 28 compounds, dominated by flavones, namely, naringenin, trihydroxy-dimethoxy flavone, and dihydroxy-trimethoxy flavone. Among others, glycosides, such as clerodendrin, calceolarioside E, forsythoside B, geniposide, and martynoside, and glucuronides, such as luteolin-7-diglucuronide, tricin-7-O-glucuronide, and luteolin-7-O-glucuronide, were also identified. *LEAE* exhibited exceptionally high *in vitro* [LC_50_ = 6,900 parts per million (ppm)] and *in vivo* (computed as damage score on a scale of 1–9) insecticidal activity against *S*. *frugiperda*, with no phytotoxicity at a dose as high as 20 times of LC_50_. *LEAE* also exhibited significant antifeedant, ovicidal, and growth regulatory activity at the 70–16,000 ppm (w/v) concentration range. *In silico* assessment revealed strong binding of martynoside, calceolarioside E, and forsythoside B with acetylcholinesterase-, sodium-, and chloride-dependent γ-aminobutyric acid (GABA) receptor and ryanodine receptor, respectively, facilitated by hydrogen bonds (conventional and C–H bonds) stabilized by hydrophobic pi–sigma, pi–pi stacked, pi–alkyl, and alkyl interactions. The present study established *L. alba* as a potential bioresource and secondary metabolite enriched *LEAE* as bioinsecticide for further product development.

## Introduction

Fall armyworm (FAW), *Spodoptera frugiperda* (J.E. Smith) (Lepidoptera: Noctuidae), is an invasive pest with a wide range of hosts originating from the Americas, inflicting significant damage primarily to maize (Rukundo et al., 2020). During its larval stage, the insect exhibits voracious feeding habits, causing substantial crop damage, particularly due to its nocturnal behavior. Being polyphagous, FAW larvae can consume approximately 353 host plants spanning over 76 plant families (Padhee and Prasanna, 2019). Although it is polyphagous, it shows a preference for plants in the Poaceae (106 taxa), Asteraceae (31 taxa), and Fabaceae (31 taxa) families, adversely affecting economically important crops such as wheat, rice, maize, sorghum, sugarcane, cotton, and various vegetables (Patel Sagarbhai et al., 2021). Maize, described as the “Queen of Cereals,” is the most severely affected and preferred host of FAW worldwide (Montezano et al., 2018). If not managed timely, the damage can be as high as 70% of the crop loss (Raghunandan et al., 2019). The entry of FAW led to nearly 1,000 metric tons of maize yield loss during the 2018–2019 period. FAW was first reported in Central and Western Africa in 2016 (Goergen et al., 2016), and it created havoc in maize crop. In May 2018, FAW was first reported in India from the state of Karnataka (Kalleshwaraswamy et al., 2018).

Control measures against *S*. *frugiperda* have led to increased use of pesticides belonging to the group of organochlorines (OCs), carbamates, organophosphates (OPs), diamides, pyrethroids, etc., resulting in the development of resistance in pest leading to reduced control efficacy of recommended pesticides (Gutiérrez-Moreno et al., 2019; Garlet et al., 2021). Injudicious use of synthetic pesticides further aggravates the problem with additional concerns of toxic residues, environmental pollution, and potential threat to non-target organisms including human beings (Kundu et al., 2021; Nasir et al., 2022). To address this, alternative methods such as plant extracts and essential oils have gained prominence for insect pest control (Krinski et al., 2014; Dutta et al., 2021).

Plants produce secondary metabolites of varying profile as natural self-defense against pests and pathogens (Lyubenova et al., 2023). The compounds produced include alkaloids, saponins, tannins, phenols, and terpenoids (Divekar et al., 2022). These secondary metabolites exhibit a multifold insect-controlling mechanism manifested as insecticidal, antifeedant, repellent, oviposition deterrent, and growth-regulating effects (Tlak Gajger and Dar, 2021; Twaij and Hasan, 2022). The utilization of plant extracts as botanical pesticides in pest management practices offers several advantages including target specificity, consumer and environment safety, reduced synthetic pesticide application, and residue-free organic produce (Lengai et al., 2020).

Among bioactive-rich plants of medicinal value, the genus *Lippia* (Verbenaceae) includes approximately 200 species of herbs, shrubs, and small trees, mainly distributed throughout the South and Central America and tropical Africa territories (Terblanché and Kornelius, 1996). In India, it is mainly distributed over the states of Andaman and Nicobar Islands, Andhra Pradesh, Assam, Bihar, Gujarat, Madhya Pradesh, Meghalaya, Odisha, Tamil Nadu, Uttar Pradesh, and West Bengal. Most of the species are traditionally utilized as gastrointestinal and respiratory remedies. *Lippia alba* (Mill.) N.E. Br., a prominent member of the genus, is primarily known for its use in treating digestive, respiratory, sedative, cardiovascular, and other miscellaneous issues (Hennebelle et al., 2008). The volatile (essential) oil and aqueous extract of *L*. *alba* leaves have been reported to have insecticidal activity against FAW (Camilo et al., 2022). However, the other non-polar and polar fractions have never been mapped in relation to insecticidal potential. The gap thus presents a promising opportunity to map the bioactive fraction against FAW and standardize the dose of the most effective fraction. Keeping this in view, the hypothesis of the present study was planned to identify the most effective fraction of *L. alba* leaves impacting insecticidal efficacy and the characterization of the responsible phyto-compounds and their correlation for inhibiting selected proteins of *S. frugiperda*.

## Materials and methods

Hexane, dichloromethane, ethyl acetate, and methanol were procured from Merck^®^ India Ltd. (Mumbai, India) for use as extraction solvents. Solvents were evaporated under reduced pressure below 45°C using a flash evaporator (Heidolph, Germany).

### Plant sample

In August 2022, fresh *L. alba* leaves (5.0 kg) were harvested from the CSIR-Central Institute of Medicinal and Aromatic Plants (CIMAP), located in Lucknow, Uttar Pradesh [26°53°37°N, 80°58°57°E 141 m above mean sea level (AMSL)]. The leaves were rinsed in distilled water followed by shade drying and ground into coarse powder. The powder was preserved in airtight polybags at 4°C.

### Extraction

The leaf powder was extracted using the solid–liquid cold extraction method with few modifications as described by Dutta and Kundu (2022). Briefly, the coarse powder sample (500 g) was sequentially extracted with solvents (500 mL × 3) of increasing polarity (hexane, dichloromethane, ethyl acetate, and methanol) followed by agitation in a shaker continuously for 4 h. To obtain the highest extraction yield, extraction was repeated three times in each solvent. *Lippia* hexane extract (*LHE*), *Lippia* dichloromethane extract (*LDE*), *Lippia* ethyl acetate extract (*LEAE*), and *Lippia* methanol extract (*LME*) were obtained by filtration and subsequent evaporation of the excess solvents using a vacuum rotary evaporator while keeping the temperature at or below 35°C. Each concentrate was weighed (mg/g of sample, dry weight basis) and kept at −20°C for chemoprofiling and bioassays.

### *In vitro* insecticidal activity

#### Rearing of insect

The neonate larvae of FAW were collected from infested maize plants in the field of ICAR-IARI, New Delhi (28°38°39°N, 77°09°09°E), as the initial culture or population. Larvae were reared in the laboratory at 28 ± 1°C and 65% ± 2% relative humidity (RH) under a 16-h light/8-h dark photoperiod initially for 5 days in a group of ~100 in round containers (15 cm diameter, 2 cm height) containing a 2- to 3-mm layer of chickpea flour–protein-based artificial diet (Singh and Rembold, 1992). Later, the larvae were transferred individually to multi-well plates with each cell being 2.5 cm in diameter and 2.3 cm in depth to avoid cannibalism and maintained until pupation. The pupae were sterilized with 2% sodium hypochlorite solution and kept in groups of 25–50 in Petri plates (20 cm diameter). After adult emergence, three pairs of FAW moths were released inside 2-L glass jars lined with bloating paper. The adult moths were provided with a 10% honey solution. The eggs were scrapped off daily on Petri plates and the neonate larvae were transferred to semi-synthetic diet for rearing. The larvae of desired instars were used for bioassay (Kumar et al., 2023).

#### Leaf-dip bioassay

Leaf extracts [*LHE*, *LDE*, *LEAE*, and *LME*; 50,000 parts per million (ppm) stock solutions] were prepared using 1% Tween 80 as solvent by stirring at 500 rpm for 30 min. Subsequent dilutions were made to obtain the test concentrations whereas 1% Tween 80 solution in distilled water was used as control. Maize leaves cut into 4.5-cm pieces were dipped into different concentrations (600–50,000 ppm) of test formulations. The experiment was set up in completely randomized design (CRD), with three replications of each treatment, each with 15 second instar larvae per replication, and each one being placed separately in 5-cm Petri plates. Larval mortality was recorded after 72 h of feeding. Based on the results of this bioassay, *LEAE* was found to be the most active extract against *S. frugiperda*. Hence, it was chosen for further efficacy studies such as antifeedant, repellent, ovicidal, and growth regulatory activities along with liquid chromatography–mass spectrometry (LC-MS) analysis and molecular docking studies.

### LC-MS analysis

The extract was phytochemically characterized using an Acquity ultraperformance liquid chromatograph (UPLC) connected to a quadrupole time-of-flight mass spectrometer (QToF-MS, Synapt G2 HDMS, Waters Corporation, Manchester, UK). Using electrospray ionization (ESI) and mass resolution nominally set at 20,000, the QToF-ESI-MS was managed using the MassLynx 4.2 software. The MSE function was employed in continuum mode to collect data in the 50–1200 *m*/*z* range. Full-scan MS data (low energy, 4 V) and MS/MS data (high energy, 10.0–60.0 V ramping) are accessible at the same time when using the MSE mode. Capillary 3 kV, sampling cone 30.0 V, extraction cone 5.0 V, source temperature 1,200°C, desolvation temperature 500°C, desolvation gas flow 1,000 L/h, and cone gas flow 50 L/h were the parameters of the source. A reference mass leucine enkephalin (*m*/*z* 556.2771 in positive polarity and *m*/*z* 554.2670 in negative polarity) was utilized for the mass correction, or lock spray, at a flow rate of 10.0 µL/min and a concentration of 2.0 µg/mL/20.0 s. Using an ACQUITY UPLC BEH C18 column (2.1 ×100 mm, 1.8 µm, Waters India Pvt. Ltd., Bangalore), chromatographic separation was performed at 35°C. The mobile phase consisted of two phases: an A phase, 10:90 methanol:water, and a B phase, 90:10 methanol:water, both containing 0.1% formic acid. Employing a 0.4 mL/min flow rate, the gradient program was run for 0–0.5 min/90% A, 0.5–4.5 min/50% A, 4.5–18.0 min/50–2% A, 18.0–20.0 min/20% A, and 20.0–25.0 min/90% A. Raw data were processed using the UNIFI software 1.7 version (Waters Corporation, Manchester, UK) and a phytochemical database, specifically focused on phenolic compounds, created in accordance with DG SANTE guidelines (Kumar et al., 2021; Dutta and Kundu, 2022).

### *In silico* molecular docking studies

Based on the chemical profiling and characterization of the most effective fraction, a total of 28 compounds were assessed through molecular docking studies for insecticidal potential against *S*. *frugiperda*. Acetylcholinesterase (AchE), ryanodine receptors (RyRs), and sodium- and chloride-dependent γ-aminobutyric acid (GABA) transporters (GATs) were chosen as targets for molecular docking analysis. These target proteins play a major role in the transmission of signal functioning in the insect nervous system (Maule et al., 2006).

#### Preparation of the 3D structure of receptors

The National Center for Biotechnology Information (NCBI) database provided the target proteins’ amino acid sequences for testing. Furthermore, the NCBI blast tool and Protein Data Bank (PDB) database were utilized to locate templates appropriate for building the secondary structures of the selected amino acid sequences. After that, homology protein structures were modeled using Modeller v. 9.24 and saved in pdb format. Using PROCHECK software, the quality of the modeled receptor protein was evaluated in order to confirm its accuracy.

#### Ligand preparation

In the context of this inquiry, the term “ligands” implies the three-dimensional (3D) molecular structures of the identified substances employed for molecular docking. Utilizing the PUBCHEM database and ChemDraw Ultra 11.0 software, the 3D structures of the compounds undergoing screening were acquired and stored as sdf file type.

#### Molecular docking

*In silico* molecular docking investigation was performed with See SAR v10.3.1. Using homology modeling, the receptor protein structures were constructed and the empty active site residues were identified. For the entire set of molecules, both 2D and 3D frameworks were developed. Following docking, the binding affinity was determined using HYdrogen DEhydration (HYDE) scoring, which takes into account the desolvation and hydration processes and is based on the molecules’ octanol–water partition coefficients (*K*_ow_). The estimated affinities of the ligands for binding the receptor proteins varied from millimolar to picomolar. Additional considerations were made for torsion quality, clashes, ligand efficiency (LE), and lipophilic ligand efficiency (LLE) in order to determine the optimal combinations. The interactions between the docked receptor and ligands were examined using Discovery Studio v4.1.

### Pest behavior study

#### Antifeedant bioassay

Maize leaf pieces (4.5 cm) were dipped into various concentrations (200–16,000 ppm) of *LEAE* and air-dried. Treated leaves were placed singly in 5-cm Petri dishes, and one second instar *S*. *frugiperda* larva was released per treated leaf. The experiment was carried out in CRD, where each treatment was replicated four times. Leaf area fed by FAW during 24 h was measured using a leaf area meter, and the data were processed using the IBM SPSS statistics 20.0 software. The antifeedant index was measured using the formula of Jannet et al. (2001).


Antifeedant Index=(C−T)∗100/(C+T)

where C and T represent the leaf area eaten by the larva on the control and treated leaf, respectively.

#### Repellent assay

*LEAE* was further evaluated for potential repellent activity via two-choice bioassay. Maize leaves (4.5 cm) treated with five concentrations of *LEAE* (200–16,000 ppm) were air-dried. Each treated leaf piece with a non-treated counterpart was placed in a Petri plate (10 cm diameter) lined with filter paper and five second instar FAW larvae were released in the center. Four replications were kept for each treatment. Repellency effect was measured at 5 min, 2 h, and 24 h by counting the larvae on the treated vs. untreated leaf piece. Repellence was calculated by the formula given by Farag et al. (2011).


Percent Reppellency=(C−T)∗100/(C+T)

where C and T represent the number of larvae present on the control and treated leaf, respectively.

#### Ovicidal effect

An additional evaluation of *LEAE’s* possible ovicidal action was conducted. The breeding cages were equipped with filter paper strips, which allowed the females to lay their eggs directly on top of the paper. Every day, the paper strips were taken off and chopped into round bits. To preserve only 30 eggs per circle, the paper circles were cleaned with a histology needle under a 40× stereoscopic microscope. Ten replicates of 30 FAW eggs (24 h old) were used to assess the extract’s ovicidal activity. The eggs were put in 4-cm^2^ circular filter paper pieces. For 10 s, eggs were submerged in five distinct extract concentrations, ranging from 500 to 16,000 ppm, as well as distilled water as the control. After that, they were moved to the filter paper and placed in a laminar flow hood for 5 min to remove any remaining moisture. Subsequently, the replications, or filter papers, containing the eggs were placed in plastic Petri dishes until the larvae emerged. The percentage of eggs that failed to hatch was used to calculate the ovicidal activity.

#### Sub-lethal effect

The effect of sub-lethal concentrations of the *LEAE* was evaluated by life table analysis using the diet incorporation method. An FAW larval diet was incorporated with different concentrations of *LEAE*, viz., 0, 0.007, 0.02, 0.06, and 0.2 mg/g of diet. The diet (1.5 g) was placed in a Petri plate and a single second instar larva (8.15 ± 1.28 mg) was released into it. Twenty such larvae were observed per treatment until adult emergence. The parameters recorded were larval weight and mortality after 7 and 14 days, pupation, deformity, and adult emergence.

#### *In vivo* assay

Plants of the well-known hybrid IMH 1308 were raised in a phytotron chamber at the National Phytotron Facility, ICAR-IARI, New Delhi, with controlled atmospheric conditions of 28°C, 65% RH, and a 14:10 photoperiod. Five seeds were sown in 9-inch pots that were filled with a sterilized mixture of soil, sand, cocopeat, and FYM in a 2:1:1:1 ratio, with four plants kept per pot. Four pots served as a replication for each of the five treatments that were applied in triplicate. After 15 days of germination, 52-cm-tall polypropylene sheets were placed over each pot, and three larvae of the second instar FAW (12 larvae/pot), with the exception of the negative control, were released into the whorls of each plant. Following a 48-h period, the whorl, surrounding leaves, and stem were treated with treatment solutions using an atomizer. There was only water in the control treatments. Following a 72-h period, damage symptoms were graded on a 1–9 scale (1 being no harm and 9 being fully damaged), and the weights of the plants were noted (PW).

#### Statistical analysis

The obtained data were analyzed using analysis of variance (ANOVA), and for laboratory bioassays, least significant differences (LSDs) at 5% significance and Tukey honestly significant difference (HSD) for pot culture tests were used to compare the means of treatments and replicates.

## Results

### Yield and bioactives of *L. alba* leaves

**Figure 1:**
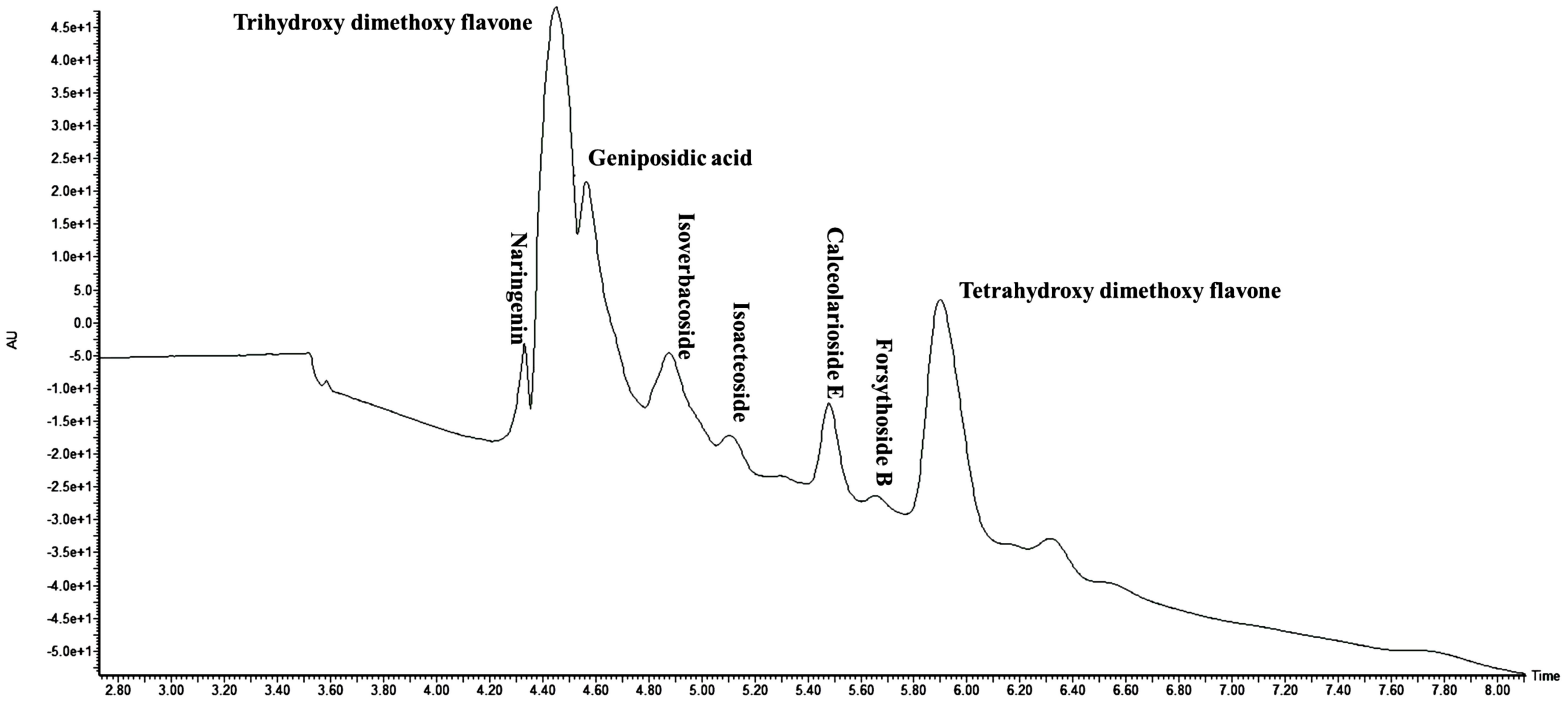
Total ion chromatogram (TIC) of *Lippia alba* leaf extract as analyzed in UPLC-QToF-ESI-MS/MS.

The yield of different extracts varied between 1.25% and 2%. The phytochemical composition of *LEAE* was determined using LC-MS analysis. A total of 28 phytochemicals primarily flavonoids, phenolics, and their glucosides were tentatively characterized from the most effective extract of *Lippia* leaves based on their accurate molecular ion peaks [M+H]^+^ detected under positive ionization mode, within the acceptable error mass value below ±10 ppm and mass fragmentation patterns (**Table 1**; **Figure 1**). Flavones, namely, naringenin (C_15_H_12_O_5_), trihydroxy-dimethoxy flavone (C_17_H_14_O_7_), dihydroxy-trimethoxy flavone (C_18_H_16_O_7_), trihydroxy-trimethoxy flavone (C_18_H_16_O_8_), hydroxy-trimethoxy flavone (C_18_H_16_O_6_), dihydroxy-dimethoxy flavone (C_17_H_14_O_6_), and tetrahydroxy-dimethoxy flavone (C_17_H_14_O_8_), were detected at the retention time (*t*_R_) 4.29–5.89 min intervals with their corresponding [M+H]^+^ peaks at *m*/*z* 273.0772, 331.0185, 345.0997, 361.0923, 330.1041, 315.0874, and 347.0763, respectively. Even the methyl ether derivative of naringenin (C_16_H_14_O_5_) was detected at *t*_R_ 5.99 min with the corresponding [M+H]^+^ peak at *m*/*z* 287.0924. Glycosides such as clerodendrin (C_27_H_26_O_17_), geniposidic acid (C_16_H_22_O_10_), acteoside (C_29_H_36_O_16_), leucosceptoside (C_30_H_38_O_15_), isoverbascoside (C_29_H_36_O_15_), calceolarioside E (C_28_H_34_O_15_), forsythoside B (C_34_H_44_O_19_), geniposide (C_17_H_24_O_10_), martynoside (C_31_H_40_O_15_), mussaenoside (C_17_H_26_O_10_), theveside (C_16_H_22_O_11_), and cistanoside F (C_21_H_28_O_13_) were identified based on their respective high-resolution exact [M+H]^+^ peak at *m*/*z* 623.1247, 375.1278, 641.2068, 639.2280, 625.2129, 611.1999, 757.2526, 389.1437, 653.2468, 391.1627, 391.1227, and 489.1607. Glucuronides, namely, luteolin-7-diglucuronide (C_27_H_26_O_18_), tricin-7-O-glucuronide (C_23_H_22_O_13_), luteolin-7-O-glucuronide (C_21_H_18_O_2_), and apigenin-7-O-diglucuronide (C_27_H_26_O_17_), were further characterized from their [M+H]^+^ at *m*/*z* 639.1169, 507.1141, 463.0862, and 463.0862, respectively. Again, loganin (C_17_H_26_O_10_), an iridoid monoterpenoid, was identified from its accurate [M+H]^+^ at *m*/*z* 391.1612 with an error mass value of 2.04 ppm and characteristic fragmentation pattern. Relatively less polar shanzhiside methyl ester (C_17_H_26_O_11_) was detected at *t*_R_ 13.06 min with its respective [M+H]^+^ peak at *m*/*z* 407.1569.

**Table 1:** Phytoconstituents of ethyl acetate extract of *Lippia alba* leaves as analyzed in UPLC-QToF-ESI-MS/MS.

Peak	*t*_R_ (min)	Proposed phytochemicals	Formula	Neutral mass (Da)	[M+H]^+^	Error (*δ*, ppm)
1	4.29	Naringenin	C_15_H_12_O_5_	272.0685	273.0772	3.29
2	6.71	Clerodendrin	C_27_H_26_O_17_	622.117	623.1247	−0.16
3	4.53	Trihydroxy-dimethoxy flavone	C_17_H_14_O_7_	330.0739	331.0185	−0.60
4	4.57	Geniposidic acid	C_16_H_22_O_10_	374.1212	375.1278	−3.19
5	4.58	Dihydroxy-trimethoxy flavone (Santin)	C_18_H_16_O_7_	344.0896	345.0997	6.66
6	4.62	Trihydroxy-trimethoxy flavone	C_18_H_16_O_8_	360.085	361.0923	−1.38
7	4.64	Hydroxy-trimethoxy flavone	C_18_H_16_O_6_	329.0947	330.1041	4.84
8	4.65	Acteoside	C_29_H_36_O_16_	640.2003	641.2068	−2.02
9	4.67	Leucosceptoside	C_30_H_38_O_15_	638.2211	639.2280	−1.40
10	4.69	Luteolin-7-diglucuronide	C_27_H_26_O_18_	638.1119	639.1169	−4.38
11	4.76	Dihydroxy-dimethoxy flavone (Pectolinarigenin)	C_17_H_14_O_6_	314.079	315.0874	1.90
12	4.81	Methoxy apigenin (Hispidulin)	C_16_H_12_O_6_	300.0634	301.0717	1.66
13	4.92	Isoverbascoside	C_20_H_30_O_12_	462.1737	463.1812	−0.64
14	4.95	Tricin-7-O-glucuronide	C_23_H_22_O_13_	506.106	507.1141	0.59
15	5.09	Luteolin-7-O-glucuronide	C_21_H_18_O_2_	462.0798	463.0862	−3.02
16	5.14	Isoacteoside	C_29_H_36_O_15_	624.2054	625.2129	−0.47
17	5.51	Calceolarioside E	C_28_H_34_O_15_	610.1897	611.1999	3.92
18	5.61	Forsythoside B	C_34_H_44_O_19_	756.2477	757.2526	−3.82
19	5.89	Tetrahydroxy-dimethoxy flavone (Spinacetin)	C_17_H_14_O_8_	346.0689	347.0763	−1.15
20	5.99	Naringenin-methyl ether	C_16_H_14_O_5_	286.0841	287.0924	1.74
21	6.19	Geniposide	C_17_H_24_O_10_	388.1369	389.1437	−2.56
22	6.6	Martynoside	C_31_H_40_O_15_	652.2367	653.2468	3.52
23	6.66	Apigenin-7-O-diglucuronide	C_27_H_26_O_17_	622.117	623.1259	1.76
24	6.69	Mussaenoside	C_17_H_26_O_10_	390.1526	391.1627	5.87
25	6.76	Theveside	C_16_H_22_O_11_	390.1162	391.1227	−3.32
26	6.83	Loganin	C_17_H_26_O_10_	390.1526	391.1612	2.04
27	6.9	Cistanoside F	C_21_H_28_O_13_	488.153	489.1607	−0.20
28	13.06	Shanzhiside methyl ester	C_17_H_26_O_11_	406.1475	407.1569	3.92

### Insecticidal bioassay

The results of the insecticidal bioassay of the different extracts obtained from *Lippia* are summarized in **Table 2**. All the extracts showed promising insecticidal efficacy against FAW with LC_50_ values ranging between 6,900 and 39,600 ppm after 48 h of treatment. However, *LEAE* showed the highest bioefficacy with an LC_50_ value of 6,900 ppm. Larval mortality was found to be dose dependent, representing increasing mortality with increasing concentration (**Supplementary Figure S1**). The bioefficacy of the most potent *LEAE* was further explored via phytotron studies to evaluate any possible phytotoxic effects of the extract even at higher concentrations as well as the efficacy of the extract to prevent whorl damage caused by FAW. The results (**Table 3**) clearly indicate that even at the highest treatment doses, the extract had no adverse effect on the plant in terms of phytotoxicity as well as plant weight as compared to control. The extract also showed promising preventive action against whorl damage caused by FAW.

**Table 2:** *In vitro* insecticidal activity of different extracts of *Lippia alba* leaves against FAW.

Extract type	LC_50_ (ppm)	95% confidence limit (%)		Slope ± SE	*χ* ^2^
		Lower	Upper		
*LHE*	36,900	2.64	6.90	0.996 ± 0.135	2.07
*LDE*	10,260	0.553	2.027	1.144 ± 0.113	4.95
*LEAE*	6,900	0.353	1.302	1.209 ± 0.113	5.65
*LME*	24,000	1.70	3.64	1.050 ± 0.126	1.66

**Table 3:** Pot culture studies of *LEAE* against FAW.

Treatments	Whorl damage score	Phytotoxicity score	Average weight of plants (g)
Control	8.00 ± 0.47^a^	1 ± 0.00	8.33 ± 0.40^b^
*LEAE* (LD_50_)	4.33 ± 0.27^b^	1 ± 0.00	11.67 ± 0.31^a^
*LEAE* (10*LD_50_)	2.67 ± 0.27^c^	1 ± 0.00	12.40 ± 0.43^a^
*LEAE* (20*LD_50_)	2.00 ± 0.00^c^	1 ± 0.00	12.9 ± 0.26^a^

a, b and c represent mean±standard error. Similar letters are nonsignificant as per Tukey HSD at p<0.05 level.

*LEAE* at the test concentrations were found to have antifeedant action against FAW. The antifeedant index varied between 25.76% and 90.27% at different concentrations (**Table 4**). The antifeedant activity can be attributed to the synergistic action of different groups of compounds present in the extract.

**Table 4:** Antifeedancy and repellency assay of *LEAE*.

Concentration (ppm)	% Antifeedance (mean ± SE)	% Repellence (mean ± SE) (after 5 min)
16,000	90.27 ± 5.08^a^	90.00 ± 8.66^a^
5,300	87.06 ± 7.29^a^	80.00 ± 10.00^a^
1,700	61.89 ± 5.41^b^	60.00 ± 14.14^ab^
500	42.76 ± 1.74^c^	40.00 ± 10.00^bc^
200	25.76 ± 2.03^d^	20.00 ± 0.00^c^

a, b, c and d represent mean±standard error. Similar letters are nonsignificant as per Tukey HSD at p<0.05 level.

**Figure 2:**
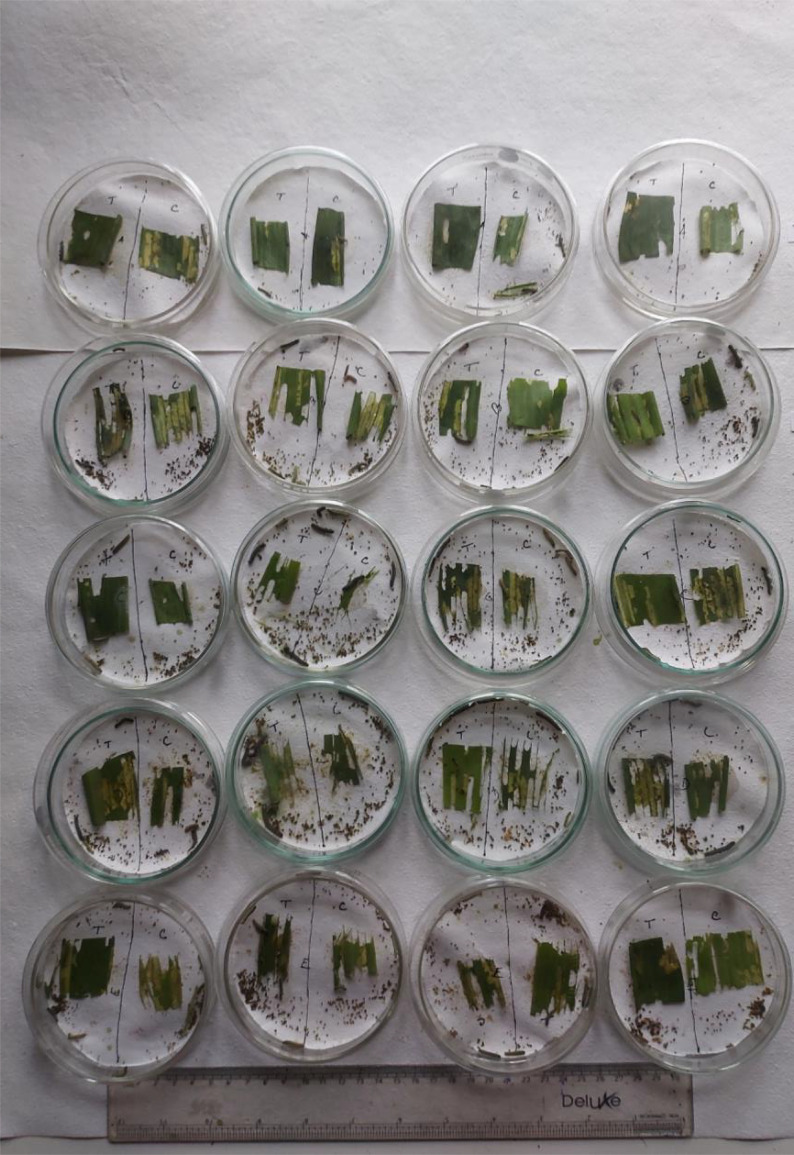
Antifeedant and repellent action shown by *LEAE* at sub-lethal concentration.

The repellent action shown by the *LEAE* was short-lived in nature. There was 20%–90% repellency after 5 min of treatment; however, after 2 h, there was high variation among the replications, and this repellence effect further diminished as time progressed and almost became non-existent after 24 h of treatment. The diminishing repellent effect can be attributed to the adaptability of FAW larva, which is a testament to its polyphagous nature since it was reported to feed on approximately 353 host plants spanning over 76 plant families (Montezano et al., 2018) (**Figure 2**).

At the test concentrations, *LEAE* showed significant ovicidal activity against FAW eggs. At the highest test concentration (16,000 ppm), 100% inhibition of egg hatching was observed after 72 h. The ovicidal activity was found to be dose dependent, as a higher number of eggs hatched as the test concentration decreased. However, even at the lowest dose, 27.78% of eggs failed to hatch after 72 h, whereas under control treatment, there was 100% egg hatching (**Table 5**).

**Table 5:** Ovicidal assay of *LEAE*.

Concentration (ppm)	% Unhatched eggs (mean ± SE)
16,000	100.00 ± 0.00^a^
5,300	86.67 ± 1.57^b^
1,700	60.00 ± 3.14^c^
500	27.78 ± 2.4^d^
0 (Control)	0.00 ± 0.00^e^

a, b, c, d and e represent mean±standard error. These letters are significantly different as per Tukey HSD at p<0.05 level.

The effect of sub-lethal doses of the *LEAE* was evaluated in terms of larval mortality, larval weight, and survival. The insects in the control group had 100% survival rate after 14 days, whereas in the case of the treatment group, it varied between 25%–85% and 0%–50% at different concentrations after 7 and 14 days, respectively (**Supplementary Table S1**). Dose-dependent larval weight reduction was also observed (**Table 6**). The extract had its impact on developmental process of the test insects as well since adult emergence was found only in the case of the control group. After 14 days, only 10%–35% larvae were in the pre-pupal and pupal stage in the case of the treatment group while all larvae reached pupal stage in the case of the control group (**Supplementary Table S1**); adult emergence was completed by the ninth day of pupation in control, whereas no adult emergence was observed in any of the *LEAE* treatments. The results indicate that higher concentrations of *LEAE* affect growth and development of FAW. Additionally, at 0.33 mg/g concentration, *LEAE* induced molting inhibition and deformity in larvae and pupae (**Supplementary Figure S2**), suggesting its growth regulatory effect.

**Table 6:** Effect of *LEAE* on larval growth.

Concentration (mg/g)	Larval weight (mean ± SE)		
	0 DAT	7 DAT	14 DAT
0.2	8.21 ± 0.28^g^	16.39 ± 0.66^g^	–
0.06	8.15 ± 0.28^g^	47.55 ± 7.38^f^	106.66 ± 11.24^de^
0.02	8.25 ± 0.29^g^	92.24 ± 6.68^e^	145.25 ± 12.06^b^
0.007	8.05 ± 0.28^g^	111.47 ± 6.77^cd^	187.69 ± 9.19a
Control	8.08 ± 0.29^g^	127.19 ± 7.94^c^	198.79 ± 8.27^a^

a, b, c, d, e, f and g represent mean±standard error. Similar letters are nonsignificant as per Tukey HSD at p<0.05 level.

### Molecular docking for the prediction of the mechanism of interaction

Three receptor proteins (putative target proteins) of *S*. *frugiperda*, namely, AchE, RyRs, and GATs, were screened against the identified major biomolecules of the most potent fraction of *L*. *alba*. Gibb’s free energy associated with the target-specific binding and other related parameters are presented in **Table 7**.

**Table 7:** Molecular docking scores associated with the protein complexes containing major components of *L*. *alba* leaves.

Target proteins	Identified compounds	Mol wt.	Log *p*	Binding affinity range	Δ*G*	LE	LLE
Acetylcholine esterase	Martynoside	652.642	−0.4098	295.945315 < KI < 29403.916812	−31.4	+	–
	8 epi-loganin	390.3824	−2.1508	2236.821954 < KI < 222241.486269	−26.5	++	–
	Naringenin	272.2548	2.5099	2724.397118 < KI < 270684.961545	−27.6	–	0
	4',5,7-trihydroxy-3,6-dimethoxyflavone	330.2906	2.4023	4735.681857 < KI < 470517.992059	−24.5	–	–
	Mussaenoside	390.3824	−2.0067	6471.634148 < KI < 642995.115901	−24	+	–
	Hispidulin	300.2648	2.4282	6838.224143 < KI < 679417.999375	−23.9	–	–
	Luteolin-7-o-glucuronide	461.3533	−1.6441	11503.798727 < KI < 1142970.419401	−16.4	+	–
	5,7,3'-Trihydroxy-3,6,4'-trimethoxyflavone	360.3164	2.4109	30713.778155 < KI < 3051595.453881	−20.1	–	–
	Spinacetin	346.2896	2.3225	98018.734403 < KI < 9738740.795548	−17.3	–	–
Sodium and chloride dependent GABA receptor	Calceolarioside E	610.5615	−1.4027	4424.750567 < KI < 439625.130017	−31.9	+	–
	Pectolinarigenin	314.2916	2.7312	14791.708282 < KI < 1469643.673361	−21.9	–	–
	Spinacetin	346.2896	2.3225	21585.783027< KI<2144675.168984	−20.9	–	–
	Cistanoside F	488.4392	−2.8514	63625.854829< KI<6321604.863112	−19.2	+	–
	5-hydroxy-3,7-dimethoxy-2-(4-methoxyphenyl) chromen-4-one	328.3184	2.9997	82062.453142 < KI < 8153389.911307	−17.8	–	–
	Hispidulin	300.2648	2.4282	131173.040824 < KI < 13032817.162265	−16.7	–	–
	Geniposide	388.3666	−2.2290	149716.107135 < KI < 14875180.435497	−16.1	+	–
	Mussaenoside	390.3824	−2.0067	155961.589383 < KI < 15495706.023104	−15.6	0	–
	Naringenin 4'-methyl ether	286.2816	2.8129	166541.10358 < KI < 16546843.309575	−16.1	–	–
	Naringenin	272.2548	2.5099	173832.006101 < KI < 17271237.582306	−15.7	–	–
Ryanodine receptor	Forsythoside B	756.7026	−2.551	0.138035 < KI < 13.714624	−50.7	++	–
	4',5,7-trihydroxy-3,6-dimethoxyflavone	330.2906	2.4023	1070.082272 < KI < 106319.000587	−28.4	–	–
	Santin	344.3174	2.7053	1071.369932 < KI < 106446.937184	−28.4	–	–
	Hispidulin	300.2648	2.4282	1772.331682 < KI < 176091.6315	−26.8	–	–
	Naringenin 4'-methyl ether	286.2816	2.8129	3294.552022 < KI < 327333.222324	−25.7	–	–
	Geniposide	388.3666	−2.2291	7915.537175 < KI < 786455.41872	−25.5	+	–
	Naringenin	272.2548	2.5099	26953.427699 < KI < 2677982.403175	−20.7	–	–
	Mussaenoside	390.3824	−2.0067	49397.200818 < KI < 4907903.960598	−17.1	+	–
	Theveside	389.3309	−4.5373	59220.213556 < KI < 5883878.354387	−18.4	++	–

**Figure 3:**
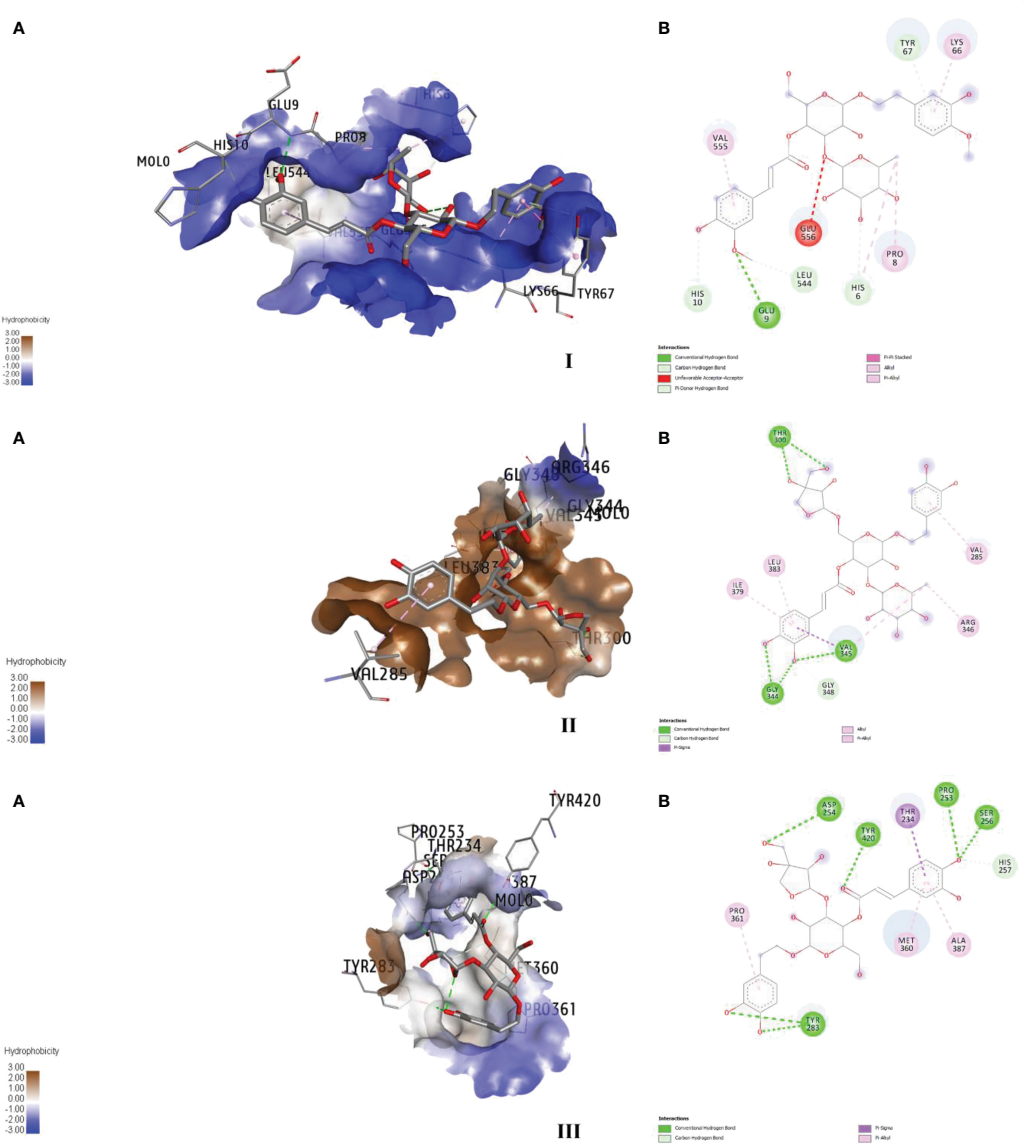
Major binding interactions **(A)** and 2D diagrams **(B)** of the ligand–enzyme complexes; martynoside–AChE (I), forsythoside B–ryanodine receptor (II), and calceolarioside E–GABA (III).

The target specific and stable favourable ligand receptor interactions are summarized in **Table 8** and **Figure 3**. In the case of AchE, martynoside was found to be the best molecule with the lowest binding energy (−31.4 kcal/mol) followed by naringenin (−27.6 kcal/mol) and loganin (−26.5 kcal/mol). This strong binding affinity can be attributed to multiple (six) H bonds (two conventional, three C–H, and one pi–donor H bond) with a shorter bond distance below 3 Å. Furthermore, LE and LLE were also calculated for selecting favorable fragments. These two parameters are directly dependent on their respective free energy of binding. Therefore, the desired positive response was observed in LE. However, martynoside is lipophobic; thus, a negative response was observed in LLE. The martynoside–AchE complex was further stabilized by the pi–alkyl and alkyl type of hydrophobic interactions.

**Table 8:** Major interactions between the protein complexes of FAW and components of *L*. *alba* leaves.

Target–ligand interaction	Interaction between	Distance	Category	Type
Sodium- and chloride-dependent GABA receptor	:SER256:OG -:MOL0:O9 :TYR283:OH -:MOL0:O8 :TYR283:OH -:MOL0:O10 :TYR420:OH -:MOL0:O1 :MOL0:O8 -:MOL0:O6 :MOL0:O9 -:PRO253:O :MOL0:O14 -:ASP254:OD2 :HIS257:CE1 -:MOL0:O9 :MOL0:C39 -:MOL0:O2 :THR234:CG2 -:MOL0 :MOL0 -:MET360 :MOL0 -:ALA387 :MOL0 -:PRO361	3.02424 2.80683 2.7488 2.95819 2.93646 2.85496 2.75823 3.21737 3.308 3.84095 4.77404 5.06986 4.79618	Hydrogen bond Hydrogen bond Hydrogen bond Hydrogen bond Hydrogen bond Hydrogen bond Hydrogen bond Hydrogen bond Hydrogen bond Hydrophobic Hydrophobic Hydrophobic Hydrophobic	Conventional hydrogen bond Conventional hydrogen bond Conventional hydrogen bond Conventional hydrogen bond Conventional hydrogen bond Conventional hydrogen bond Conventional hydrogen bond Carbon hydrogen bond Carbon hydrogen bond Pi–sigma Pi–alkyl Pi–alkyl Pi–alkyl
Acetylcholine esterase	:GLU9:N -:MOL0:O2 :MOL0:O11 -:MOL0:O13 :HIS6:CD2 -:MOL0:O12 :HIS10:CA -:MOL0:O10 :MOL0:C44 -:LEU544:O :TYR67:OH -:MOL0 :TYR67 -:MOL0 :MOL0:C46 -:PRO8 :HIS6 -:MOL0:C46 :MOL0 -:VAL555 :MOL0 -:LYS66	2.87436 3.10933 3.26326 3.2246 3.58556 3.61682 4.64764 3.99536 5.16587 4.75782 4.74679	Hydrogen bond Hydrogen bond Hydrogen bond Hydrogen bond Hydrogen bond Hydrogen bond Hydrophobic Hydrophobic Hydrophobic Hydrophobic Hydrophobic	Conventional hydrogen bond Conventional hydrogen bond Carbon hydrogen bond Carbon hydrogen bond Carbon hydrogen bond Pi–donor hydrogen bond Pi–pi stacked Alkyl Pi–alkyl Pi–alkyl Pi–alkyl
Ryanodine receptor	:THR300:OG1 -:MOL0:O13 :THR300:OG1 -:MOL0:O19 :MOL0:O9 -:GLY344:O :MOL0:O9 -:VAL345:O :MOL0:O11 -:GLY344:O :GLY348:CA -:MOL0:O9 :VAL345:CG2 -:MOL0 :MOL0:C53 -:VAL345 :MOL0:C53 -:ARG346 :MOL0 -:ILE379 :MOL0 -:LEU383 :MOL0 -:VAL285	2.76171 2.76001 2.8348 3.01663 2.96917 3.46459 3.4603 4.49681 3.99882 5.47692 4.73202 5.11794	Hydrogen bond Hydrogen bond Hydrogen bond Hydrogen bond Hydrogen bond Hydrogen bond Hydrophobic Hydrophobic Hydrophobic Hydrophobic Hydrophobic Hydrophobic	Conventional hydrogen bond Conventional hydrogen bond Conventional hydrogen bond Conventional hydrogen bond Conventional hydrogen bond Carbon hydrogen bond Pi–sigma Alkyl Alkyl Pi–alkyl Pi–alkyl Pi–alkyl

Similarly, the strong bonding in the GATs–calceolarioside E complex with a binding energy of −31.9 kcal/mol was due to multiple (nine) H bonds (seven conventional and two C–H bonds), further strengthened by the pi–sigma and pi–alkyl type of hydrophobic interaction. Herein, a positive interaction was noticed in LE, and LLE remained negative. It is noteworthy to mention that forsythoside B bound strongly with the RyRs protein complex, which was further stabilized by the six H bonds (five conventional and one C–H) along with hydrophobic interactions such as pi–sigma, alkyl, and pi–alkyl interactions. The estimated energy required for binding was −50.7 kcal/mol, with the desired LE and LLE being highly positive and negative, respectively.

## Discussion

The tremendous biofunctional efficacy of *L. alba* has been evidenced from its literature reports, suggesting exploitation of its valuable components as potential natural products in the area of crop protection research (Camilo et al., 2022). Although the plant has been investigated extensively, sufficient information on comprehensive chemical profiling is yet to be explored. Therefore, in the present study, bioactive components of the plant has been demonstrated. Ultraperformance liquid chromatography–quadrupole time-of-flight–mass spectrometric (UPLC-QToF-MS) analysis of ethyl acetate fraction (*LEAE*) revealed 28 diverse phytochemicals dominated by flavones, namely, naringenin, trihydroxy-dimethoxy flavone, and dihydroxy-trimethoxy flavone. These results are in line with a previous study by Teixeira de Oliveira et al. (2018), where flavonoids and phenolic acids were detected by HPLC-DAD analysis in the ethyl acetate extract of *L*. *alba* leaves. In the present study, glycosides such as clerodendrin, calceolarioside E, forsythoside B, geniposide, and martynoside were also identified in *LEAE*. Some of these compounds have been previously detected in the ethanolic fraction of *L*. *alba* leaves, where a variety of flavonoids have been detected via HPLC-ESI-MS analysis along with calceolarioside E, acteoside, and isoacteoside (Trevisan et al., 2016). Again, the aqueous decoction of *L*. *alba* leaves has been reported to contain two glucuronides (tricin-7-O-diglucuronide and chrysoeriol-7-O-glucuronide) and a phenylepropanoid glycoside (Timóteo et al., 2015). Our results on the characterization of glucuronides, namely, luteolin-7-diglucuronide, tricin-7-O-glucuronide, and luteolin-7-O-glucuronide, have been corroborated by the previous report documenting the occurrence of glucuronides in *Lippia*.

Phytochemical rich extracts of *Lippia* sp. have been reported to possess insecticidal activity against a wide range of insects (Correia-Oliveira et al., 2012; Napal et al., 2015). The traditional use of the genus *Lippia* sp. as natural pesticide has been well documented in literature (Camilo et al., 2022). In the present study, *Lippia* leaf extracts showed significant larval mortality against FAW. The insecticidal potential of the plant has been attributed to the phytochemicals belonging to alkaloids, flavonoids, phenols, tannins, and saponins (Sivira et al., 2011; Flores et al., 2017). In the recent past, Phambala et al. (2020) reported the *in vitro* insecticidal activity of the *L. javanica* aqueous extract against FAW by contact toxicity and feeding assays at the rate of 10% (w/v), resulting in 66% and 62% larval mortality, respectively. Similarly, Figueroa Gualteros et al. (2019) reported the positive effect of the *L*. *alba* aqueous extract under field conditions against FAW. However, another plant, *Vitex polygama* (Verbenaceae), has also been reported to show impressive efficacy with the application of its hydroalcoholic extract from both leaves and fruits exhibiting complete larval mortality after 72 h at the lowest concentration of 1 mg/g (Gallo et al., 2006).

In addition to the contact toxicity, *LEAE* had good antifeedant, ovicidal, and growth regulatory activities. Plant extracts have been previously reported to possess good antifeedant activity against FAW. For example, the antifeedant activity of *Jatropha gossypifolia* and *Melia azedarach* leaf extracts has been reported by Bullangpoti et al. (2012) along with their synergistic effect on cypermethrin. Citrus limonoids and their semisynthetic derivatives have also been reported to have promising antifeedant activity against FAW larvae (Ruberto et al., 2002). In a previous study, the stem and leaf extracts of *Psychotria* spp. have been found to be ovicidal on FAW besides exhibiting egg hatching inhibition ranging between 70.14% and 94.44% (Tavares et al., 2013). In another study, the methanolic extract of *Copaifera langsdorffii* leaf, bark, and fruit peel incorporated via diet resulted in reduced larval growth, longer pupal duration, and lower fertility and fecundity (Alves et al., 2012). In the same year, Salinas-Sánchez et al. (2012) reported that the acetone extract of *Tagetes erecta* (500 ppm) caused 50% reduction of larval weight and 40%–80% pupal mortality against FAW.

Studies on molecular modeling and interaction with the target-specific protein has gained immense importance recently for explaining the mechanism of interaction between the ligands and target proteins. Previously, molecular docking studies revealed that terpenoids and flavonoids have been efficient to bind favorably with the AchE enzyme of FAW (Herrera-Mayorga et al., 2022; Firdausiah et al., 2023). Similarly, the components from essential oil have been known to bind with various target sites including AchE and GABA receptor (Usseglio et al., 2022). In our study, *in silico* studies have been performed to see the site of action, revealing the strong binding of martynoside, calceolarioside E, and forsythoside B with AchE, sodium- and chloride-dependent GABA transporters, and RyR, respectively, facilitated by hydrogen bonds (conventional and C–H bonds) stabilized by hydrophobic interactions such as pi–sigma, pi–pi stacked, pi–alkyl, and alkyl interaction. The multi-target action of forsythoside, martynoside, and calceolarioside, resulting in insecticidal activity, has also been reported previously (Loza-Mejía et al., 2018). Forsythoside A and calceolarioside A–E have been found to bind strongly with ecdysone receptor (EcR) and AChE. Furthermore, calceolarioside A, C, and D have been docked strongly with prophenoloxidase (PPO). Likewise, martynoside showed insecticidal activity as well as AchE inhibitory activities (Cespedes et al., 2013). In the present study, favorable interactions have been confirmed between *LEAE* phytochemicals and the target enzymes, possibly contributing to its insecticidal action against FAW.

## Conclusion

Plants having pesticidal properties can prove to be a suitable alternative to toxic synthetic pesticides in terms of efficacy, environmental safety, and sustainability. In the present study, the ethyl acetate extract of *L*. *alba* leaves showed potent insecticidal action under both *in vitro* and controlled conditions without having any phytotoxic effect. In addition to the lethal action, the extract was also found to have promising antifeedant, ovicidal, and growth regulatory activities attributed to the diverse group of phytochemicals as analyzed by UPLC-QToF-MS. *In silico* studies revealed strong binding of the selected phytochemicals of *L. alba* with AchE, GABA, and RyR target sites of FAW. The study confirms the potential of *L*. *alba* for the management of FAW. However, it needs to be formulated suitably before further establishing the insecticidal activity by multilocational field trials.

## Data availability statement

The original contributions presented in the study are included in the article/**Supplementary Material**. Further inquiries can be directed to the corresponding author.

## Ethics statement

The manuscript presents research on animals that do not require ethical approval for their study.

## Author contributions

SB: Data curation, Formal analysis, Investigation, Methodology, Validation, Writing – original draft. AdK: Formal analysis, Writing – review & editing. SBS: Investigation, Validation, Writing – review & editing. ASK: Software, Writing – review & editing. NP: Formal analysis, Writing – review & editing. AKS: Resources, Writing – review & editing. RV: Resources, Writing – review & editing. SS: Writing – review & editing. AM: Formal analysis, Software, Writing – review & editing. TB: Supervision, Writing – review & editing. AnK: Data curation, Supervision, Writing – review & editing. AS: Conceptualization, Funding acquisition, Resources, Supervision, Writing – original draft, Writing – review & editing, Project administration.
