# Supplementary material for: Lippia alba—a potential bioresource for the management of Spodoptera frugiperda (Lepidoptera: Noctuidae)
Source: Front Plant Sci. 2024 Aug 8;15:1422578. doi: 10.3389/fpls.2024.1422578 (PMC11338851; doi:10.3389/fpls.2024.1422578)
Supplement: Supplementary file 1 [file DataSheet_1.docx]

Table S1: Sub-lethal impacts of *LEAE* on FAW

| **Concentration (mg/g)** | **% Mortality** | | **Pupation % (14 DAT)** |
| --- | --- | --- | --- |
|  | **7 DAT** | **14 DAT** |  |
| 1 | 75 | 100 | - |
| 0.33 | 25 | 55 | 15 |
| 0.11 | 20 | 55 | 15 |
| 0.04 | 15 | 50 | 25 |
| Control | 0 | 0 | 100 |


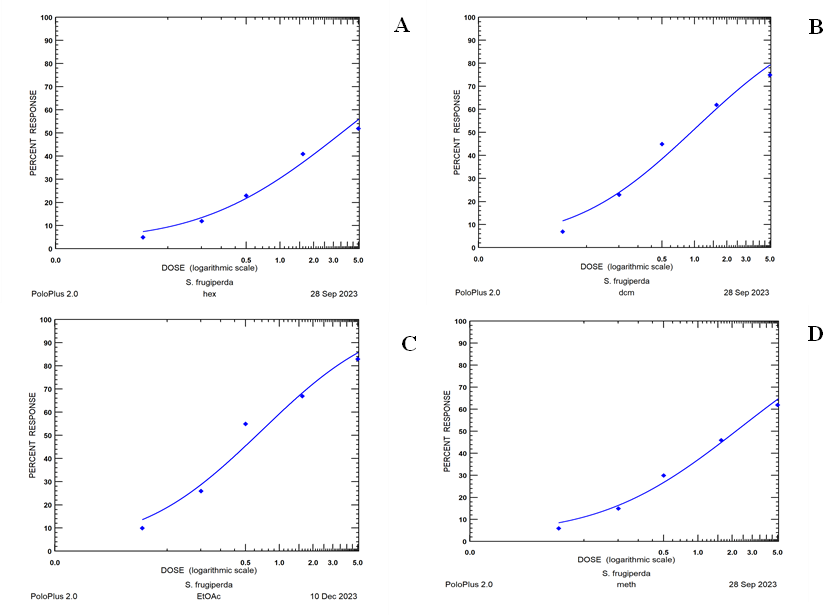


Figure S1: Dose response curve of different extracts of *L*. *alba* leaves against FAW


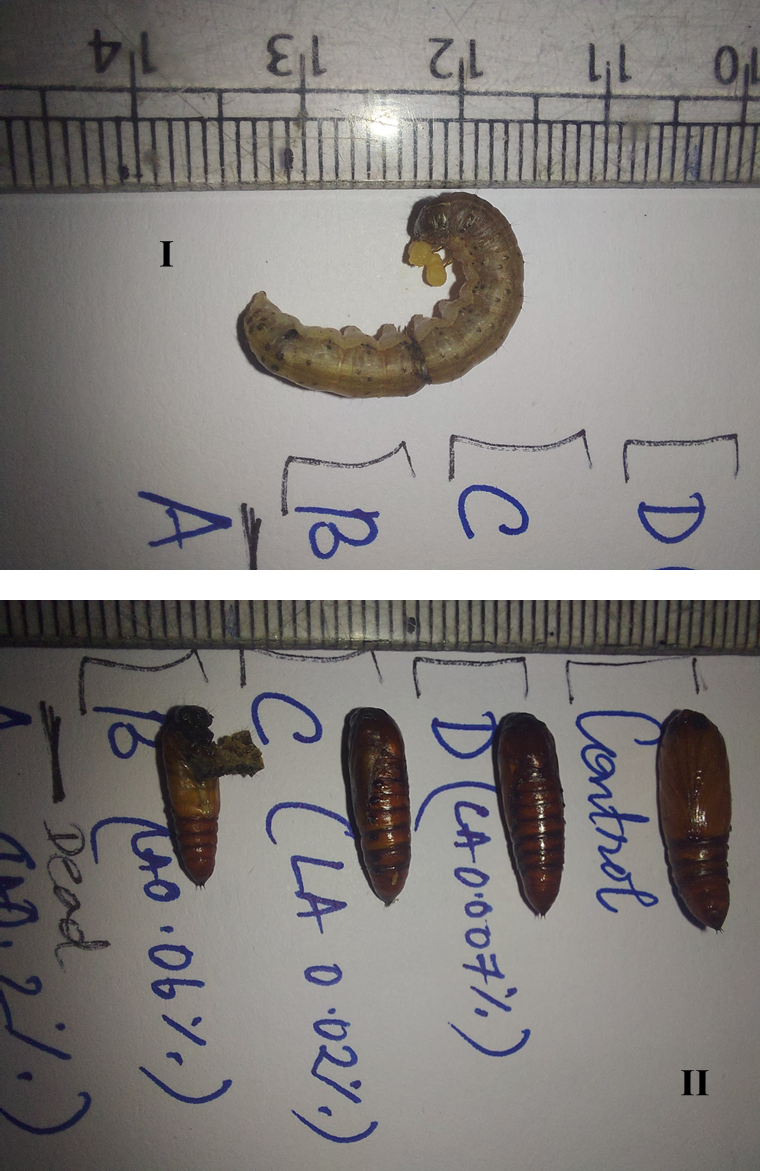


Figure S2: Larval (I) and pupal (II) deformities showed by *LEAE* at sub-lethal concentrations
